# Heat Acclimation Knowledge among Recreational Runners

**DOI:** 10.3390/sports11020049

**Published:** 2023-02-20

**Authors:** Alexander J. Heatherly, Jennifer L. Caputo, Samantha L. Johnson, Dana K. Fuller

**Affiliations:** 1 Department of Biology, Ave Maria University, Ave Maria, FL 34142, USA; 2 Department of Health and Human Performance, Middle Tennessee State University, Murfreesboro, TN 37132, USA; 3 Department of Psychology, Middle Tennessee State University, Murfreesboro, TN 37132, USA

**Keywords:** running, heat, acclimation, humidity, heat index, apparent temperature

## Abstract

Heat acclimation (HA) is the foremost method of preventing exertional heat illness during exercise in hot and humid environments. However, the prevalence of HA training and its associated knowledge is not currently known in recreational running populations. The purpose of this study was to determine the knowledge of recreational runners toward HA. A survey consisting of 38 questions that required approximately 10–15 min to complete was disseminated to running clubs throughout the Southeastern United States. Questions were designed to collect data on participant demographics, yearly training habits, and HA knowledge. Recreational runners (*N* = 125) demonstrated a lack of knowledge toward proper HA training and its associated benefits. Participants largely received HA advice from their peers (31.2%) and reported no professional guidance in their training (79.2%). Finally, participants’ beliefs toward proper HA training differed among training groups with moderate and high groups perceiving greater frequency, miles/wk, and min/wk as appropriate for HA compared to the low group (*p* ≤ 0.05). Due to the warmer temperatures and higher relative humidity experienced in the southeastern, southwestern, and mid-Atlantic locations of the United States and throughout certain regions of the European Union, governing bodies in sport and exercise science should develop more educational initiatives to convey the importance and advantages of HA, especially when runners are training for major marathons that are typically held in the late spring and early fall seasons.

## 1. Introduction

Exertional heat illness, among individuals performing physical activity in thermally stressful environments, is especially prevalent in the Southeastern United States, in which average maximum ambient temperatures of >30° and relative humidities ≥85% are experienced throughout the summer season [1,2,3]. Exertional heat illness encompasses multiple health conditions that manifest with symptoms such as headache, nausea, and vomiting. Heat stroke and heat exhaustion are forms of exertional heat illness with core temperatures of <40 °C and >40 °C, respectively [4].

The National Athletic Trainers Association recommends certified athletic trainers provide heat acclimation (HA) programs to athletes to reduce the occurrence of exertional heat illness [5]. Heat acclimation results in physiological adaptations to hot and humid environments via repeated exposure, leading to better management of internal and external heat stress [6]. Although various methods of HA exist, the physiological adaptations are similar, namely the greater dissipation of heat caused by an increased plasma volume and sweat rate, leading to greater potential evaporative cooling [7]. Athletes often utilize HA training techniques in the weeks leading up to competitions in thermally stressful environments [8].

Professional athletes often have educated exercise science professionals assisting with performance enhancement and HA training protocols [9]. Conversely, recreationally active individuals may not have access to this same expertise. Hosokawa et al. (2019) determined in a sample of 2091 recreational runners, only 47.4% were aware of the correct duration of a HA protocol [9]. Additionally, Shendell et al. (2010) [10] sampled 1138 recreational marathon runners in the Southeastern United States and found 47.9% did not understand the risks of dying from heat stroke.

Recreational athletes were found to receive a large portion of their hydration advice from peers, and it is possible that the same trend may occur for HA [11]. Furthermore, multiple studies across recreational to elite endurance athlete populations have reported that >40% of athletes surveyed experienced symptoms of exertional heat illness [8,11,12,13]. With the prevalence of exertional heat illness among recreational athletes, in addition to data showing both college and even professional athletic populations often engaging in physical activity in dehydrated states, it is important that athletes are made aware of strategies to mitigate heat stress and reduce the risk of exertional heat illness during physical activity [14,15,16,17,18,19,20]. Additionally, it is especially important to educate the recreational athlete population as it could be speculated that these individuals are at a greater risk of injury due to lack of guidance from sports medicine professionals. If this population lacks knowledge in the realm of HA strategies, more educational material and initiatives are needed for the recreational athlete. Thus, the purpose of this study was to assess the recreational running population to determine their knowledge of proper HA protocols, its associated benefits, and the sources from which they receive HA information. A secondary purpose was to determine if any differences in HA knowledge were present among recreational runners of various training categories. The current authors hypothesized that runners categorized into a “low” training category based on their training preferences would demonstrate significantly less overall training in the measured variables (frequency, volume, etc.) for each season when compared to runners categorized into a “high” training group.

## 2. Materials and Methods

### 2.1. Participants

Participants were recruited via email lists for local running clubs in the Southeastern United States (U.S.) as well as by word of mouth with an electronic announcement that included a link to the survey on a hosting website (Qualtrics, Provo, UT, USA). Inclusion criteria included males and females between the ages of 18 and 70 years, running at least 3 times per week for at least 1 year and living and training in the Southeastern U.S. For this study, the states of South Carolina, North Carolina, Tennessee, Mississippi, Alabama, Georgia, and Florida were considered to make up the Southeastern U.S., based on Diem et al. (2017) [21]. Arkansas and Louisiana were additionally included based on their status as a humid sub-tropical climate [22]. All procedures were explained in an informed consent form at the start of the electronic survey. Participation was voluntary and participants were free to withdraw from the study at any time.

### 2.2. Questionnaire

The questionnaire included modified or adapted questions from previously used questionnaires along with original questions specific to the population being surveyed [8,9,10,11,12,23,24]. The survey included 38 questions and required approximately 10–15 min to complete. The questionnaire included five sections: demographics, yearly training habits, HA and related topic knowledge, HA practices during the summer, and experiences with exertional heat illness. This manuscript includes results from the first three sections of the survey with a focus on participant demographics, seasonal training, and HA knowledge.

Section one of the survey contained nine questions on age, sex, education level, profession, geographic location, training and racing history, and participants’ predicted current 10 km time based on their current training practices and their most recent race finishing time, if applicable. Section two included 12 questions on training practices throughout the year split into three questions per season. Specific questions included running intensity on a 0–10 rating of perceived exertion scale [25], running duration, as well as frequency, mileage, and running minutes per week. Participants were asked to report weekly training in miles due to the sample being from the United States, wherein the imperial measurement system is used. The current researchers converted the data to km/wk for international reporting purposes. Section three of the survey included eight questions on participants’ HA knowledge. Specific questions were asked regarding where HA information was acquired, past attempts at engaging in HA protocols, perceived appropriate training frequency, exercise duration, exercise intensity, perceived appropriate training times during a summer day, the benefits of HA, and whether participants’ consistently ran during the hottest part of the day during the summer in order to become heat acclimated.

### 2.3. Statistical Analysis

All data were collected via Qualtrics software (Qualtrics, Provo, UT, USA) and imported to SPSS version 27.0 (IBM Corp., Armonk, NY, USA) for analysis. Means ± *SD*s are reported for quantitative demographic variables and frequencies and percentages are reported for qualitative demographic variables. Participants were divided into low and high training groups based on a product of summer training frequency, summer miles/wk, and predicted 10 km race time to determine if there were any differences in HA knowledge based on training status. Welch’s *t* tests were performed on the quantitative responses and chi-square tests of independence were performed on the qualitative responses to determine differences among training groups. An alpha of ≤0.05 was used for all analyses.

## 3. Results

A total of 216 surveys were collected with 125 of them meeting the inclusion criteria (*N* = 125). Responses from individuals older than 70 years of age or from individuals who did not train and reside in the Southeastern United States were excluded from the analyses. The final sample resulted in an approximate power of 0.80 for a medium effect size and alpha 0.05 for independent *t* tests. The final sample included 55 males and 70 females with an average age of 44.6 years ± 12.2 years.

### 3.1. Demographics

The participants in this study were experienced recreational runners with 14.1 ± 11.5 years of training experience, 13.1 ± 11.3 years of race experience, and reported a predicted 10 km race time of 55.8 ± 13.3 min. Training status of the participants constituted a total 64 in the low training group category and 61 in the high training group category. Table 1 and Table 2 provide details on geographic location and type of professional training guidance received, respectively. Almost half (46.4%) of the participants reported having earned a graduate degree, while 40% of the participants reported holding a bachelor’s degree, and 4.8% reported holding an associate degree. The remaining participants reported other forms of education (graduate school students, some college education, and high school diploma).

### 3.2. Yearly Training Habits

Table 3 includes training habits across different seasons for participants of low and high training groups. In Table 3, the significant differences found for duration, frequency, mileage (reported in kilometer units), and minutes/week within each season are presented. The low training group ran with less duration, frequency, km/wk, and min/week than the high training group for each season.

### 3.3. Heat Acclimation Knowledge

The first question of Section 3 in the survey was “What are the benefits of a heat acclimation protocol? Check all that apply.” Participants were required to correctly identify HA benefits out of seven options, with four options being true and three options being false. A total of 3.2% (*n* = 4) of participants identified three options correctly, 30.4% (*n* = 38) identified four options correctly, 28.0% (*n* = 35) identified five options correctly, 27.2% identified six options correctly, and 11.2% (*n* = 14) of participants identified all seven options correctly. The Welch *t* test revealed no statistical differences in the number of correct responses among the low (*M* = 5.1) and high (*M* = 5.2) training groups (*M*_Δ_ = −0.07, 95% CI [−0.45, 0.31]).

The second question of Section 3 asked participants “Should runners attempt to follow a heat acclimation protocol by increasing running intensity, frequency, or duration in the summer (20 June–19 September)?” There was an approximately equal split among the participants, with 64 (51.2%) respondents answering “yes” and 61 (48.8%) answering “no.” The chi-square test of independence reported no associations between the participants belief on whether a HA protocol should be followed and training status (*N* = 125; χ^2^ = 2.9; *p* = 0.09). The third question of Section 3 asked participants “Should runners consistently exercise in the hottest part of the day during the summer?” An overwhelming majority of respondents (90.4%) answered “no”, while the remaining (9.6%) answered “yes”. The chi-square test of independence indicated no association between training status and time of day to train for HA purposes (*N* = 125; χ^2^ = 1.7; *p* = 0.19).

The final question in Section 3 asked participants “What time of day would you consider most appropriate to run at when attempting to heat acclimate?” The most common times for training were 9:00 a.m.–11 a.m. (28.8%; *n* = 36), before 8:00 a.m. (24%; *n* = 30), 3:00 p.m.–5:00 p.m. (23.2%; *n* = 29), and 6:00 p.m.–8:00 p.m. (8.8%; *n* = 11), respectively. The chi-square test of independence noted no association between time of day for training and training status (*N* = 125; χ^2^ = 1.84; *p* = 0.77).

### 3.4. Heat Acclimation Training Beliefs

**Figure 1:**
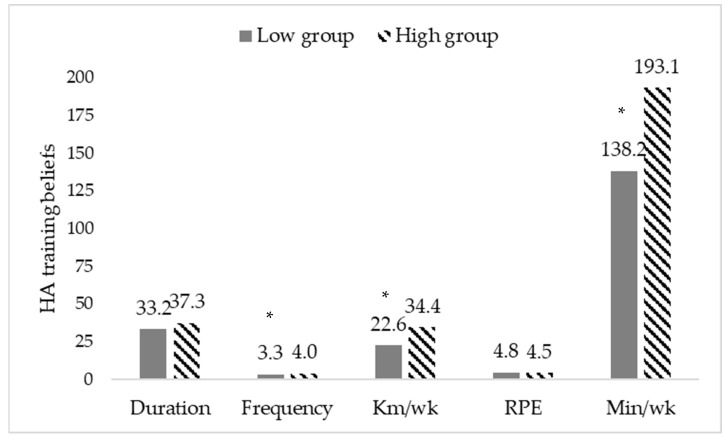
Participants’ perception of appropriate HA training during the summer. Note. * Denotes significant differences vs. the high training group at the *p* < 0.05 level. Duration = min/run, Frequency = days/wk, RPE = rating of perceived exertion on a 0–10 scale. at the *p* < 0.05 level.

Figure 1 presents the participants’ perceptions on appropriate HA training protocols for the variables of duration, frequency, km/wk, and rating of perceived exertion (RPE) during the summer season.

#### 3.4.1. Duration

Results from the Welch *t* test revealed no significant differences in perceived duration to be appropriate for HA between the low training (*M* = 33.2) and high training (*M* = 37.3) groups (*M*_Δ_ = −4.1, 95% CI [−8.9, 0.6]).

#### 3.4.2. Frequency

Results from the Welch *t* test revealed that runners in the low training group (*M* = 3.3) perceived training fewer days in a week to be appropriate for HA compared to the high training group (*M* = 4.0), (*M*_Δ_ = −0.7, 95% CI [−1.1, −0.4]).

#### 3.4.3. Kilometers

Results from the Welch *t* test revealed that runners in the low training group (*M* = 22.6) perceived fewer km/week to be appropriate for HA compared to the high training group (*M* = 34.4), (*M*_Δ_ = −11.8, 95% CI [−16.6, −7.0]).

#### 3.4.4. Rating of Perceived Exertion (RPE)

Results from the Welch *t* test revealed no significant differences in RPE between the low training (*M* = 4.8) and high training (*M* = 4.5) groups (*M*_Δ_ = 0.3, 95% CI [−0.3, 0.9]).

#### 3.4.5. Min/wk

Results from the Welch *t* test revealed that runners in the low training group (*M* = 138.2) perceived running fewer min/week to be appropriate for HA compared to the high training group (*M* = 193.1), (*M*_Δ_ = −54.9, 95% CI [−92.3, −17.6]).

## 4. Discussion

The purpose of this study was to determine the HA knowledge of a recreational running sample and to identify differences in knowledge among runners of varying training status. A sample of 125 (males = 55, females = 70) participants from the Southeastern United States responded to a survey consisting of 29 questions on training practices, HA knowledge, and HA information resources. Respondents were aged 44.6 ± 12.2 years and had an average training and racing experience of over a decade. Participants were primarily located in Alabama and Tennessee (see Table 1). Most runners in the sample reported training under no professional supervision (*n* = 99; 79.2%; see Table 2).

Most of the runners in the sample reported receiving HA advice from peers (*n* = 36; 31.2%), magazines, books, or online articles (45.6%), or not having received any information regarding HA (*n* = 48; 38.4%; see Table 2). As expected, significantly lower values for seasonal training habits were found based on training status with the low training group training less frequently (days/week), with less volume (km/week), fewer total minutes per week, and with less duration than the high training group (see Table 3).

While 30.4% of participants correctly identified approximately half of the true and false statements related to HA benefits, only 11.2% of participants correctly identified all seven true and false HA benefit statements. These findings were further reflected in the participants opinions on HA protocols, with almost half (48.8%) of the runners responding that runners should not follow an HA protocol during the summer season. Significant differences were present by training status, with the low group perceiving less training frequency, km/wk, and min/wk as appropriate for HA purposes (see Figure 1).

The current study demonstrates a lack of HA knowledge in the Southeastern recreational running population. This is especially concerning since the rate of heat-related illness is higher in the Southeastern United States compared to other regions of the country [1]. Because thermoregulatory ability is at least partially a result of aerobic fitness level, recreational running populations may be at higher risk of exertional heat illness compared to elite endurance athletes. Furthermore, with this population receiving HA information from their peers, and possibly not being guided by an exercise professional, it is hard to gauge whether scientifically accurate information is being disseminated.

The participants in the current study are comparable to those surveyed in previous studies by O’Neal et al. (2011) and Davis (2018) regarding age, training frequency, and weekly mileage [11,12]. However, the current sample was older with more training experience when compared to the previously mentioned studies. Another difference in the current study was the race distance for which the participants were asked to estimate their finishing time. Participants in the current study were asked to estimate their current 10 km finishing time while those in O’Neal et al. (2011) were given a list of ranges to choose for both half-marathon (21.1 km) or marathon (42.2 km) distances. This decision was made in order to capture the biggest sample possible, as a greater percentage of recreational runners have possibly completed this distance as opposed to longer distances (i.e., half marathon and marathon). It is important to note, however, that in the current study, the 10 km race times were self-reported predictions made by the participants based on their current training or most recent race time. The authors acknowledge that it is possible that these self-reported times may be under- or overexaggerated. It is recommended that future studies attempt to collect race times of participants directly from the entity that organized the event or test the participants via a 10 km time trial for more accurate data.

The trends regarding training volume were similar to those reported by O’Neal et al. (2011; 13.2–39.5 miles) and Davis (2018; 18.7–62.4 miles) [11,12]. Both past studies split participants into training groups based on various training-related variables and lesser trained participants reported less training volume compared to their counterparts with a higher fitness status.

The finding in the current study that 79.2% of runners did not train under professional supervision is not surprising and agrees with past studies showing that recreational runners predominantly train without professional supervision [11,12]. This finding was further reflected in the responses regarding HA information sources. The three most common responses from respondents about sources of HA information in the current study were receiving advice from peers (31.2%), magazines, books, or online articles (45.6%), or receiving no advice at all (38.4%). Similar results were found in the studies of Davis (2018), O’Neal et al. (2011), and Yates et al. (2018) with a large portion of runners reporting that advice from their peers was deemed highly important [11,12,19].

Further expounding upon the current findings that recreational runners do not train under professional supervision and receive a large portion of information from their peers, it is not surprising that only 30.4% of participants correctly identified at least half of the true and false HA statements. The lack of HA knowledge was lower than that reported by Hosokawa et al. (2019), where only 47.4% of runners that were surveyed correctly identified an appropriate timeline to attain HA [9]. Furthermore, only 51.5% of runners agreed that a person is more susceptible to exertional heat stroke in hot/humid environments. Others have also witnessed a lack of understanding in runners of the ramifications of experiencing heat stroke. Shendell et al. (2010) asked runners competing in a marathon in Atlanta, Georgia, for their beliefs on the chances of dying after experiencing heat stroke. The recreational runners (47.9%) did not believe that the chances of death were over 20% [10]. The results of the current study, taken together with those of past studies, suggests that there is a lack of information regarding HA, how it can help prevent exertional heat illness, and the health implications of experiencing this condition. Whether the findings of the current study were due to misinformed peers or inaccurate sources of information is beyond the scope of this study.

A recent study by Marocolo et al. (2021) found that of 33 social media accounts in Brazil providing exercise and training information, reaching an average of 30 million people, only 2.7% of their social media posts provided a peer-reviewed scientific citation [26]. While many print or online resources may provide scientifically sound information, the current authors caution recreational runners who receive information from sources that do not provide scientific citations and from individuals who do not hold specific educational credentials needed to provide advice on HA.

Past data indicate that more than 50% of personal trainers do not receive training information from reputable sources [27,28]. Bennie et al. (2017) also showed that of 1185 fitness professionals that were surveyed, 56% received information from other fitness professionals and 62.5% developed their own ideas toward training. It has also been reported that while 79.3% of certified athletic trainers partially follow HA guidelines, only 3.9% fully follow guidelines [29]. Combined with the findings of the current study, it is possible that there is a lack of overall knowledge toward HA among fitness professionals, and this may be a significant contributing factor to HA misinformation among recreational populations.

In the current sample, approximately half of the participants reported HA training was appropriate during the summer season. When asked their beliefs on how a runner should become heat acclimated, the training groups differed in their opinions with the low group believing less frequency, km/wk, and min/wk as more appropriate compared to the high group. Current HA guidelines recommend that exercise sessions last at least 60 min/day, have an intensity that produces an elevated core and skin temperature and causes the athlete to sweat, and lasts for seven to fourteen days [30]. The two training groups in the current study failed to meet these guidelines when reporting their beliefs on an appropriate HA protocol, except for their answers regarding exercise intensity. While the explanation for this particular outcome is open to speculation, the current authors acknowledge that variables such as a small sample size, differences in training habits, nutritional and hydration habits, or experience with past heat-related illness, or even HA could have had an influence. Future studies should attempt to collect data on these variables in an attempt to better explain if there are differences for meeting HA guidelines between runners of varying training levels.

Overall, these findings are important to note when examining exertional heat illness incidence in recreational running populations. O’Neal et al. (2011) found that 45% of recreational runners experienced exertional heat illness symptoms in the past [11]. Recreational runners are of lower fitness status than elite endurance athletes who have greater thermoregulatory abilities. The lack of HA knowledge found in the current study and exertional heat illness incidence rates found in the past, combined with a lower aerobic fitness level within this population, could pose a greater risk of exertional heat illness development when exercising in hot/humid environments.

## 5. Conclusions

The current study demonstrated that recreational runners in the Southeastern United States have a lack of knowledge toward HA and its associated benefits. Recreational runners were likely to receive information regarding HA training from their peers and sources that may not be scientifically accurate. This lack of knowledge is potentially dangerous when considering the summer training habits in hot/humid environments this population engages in and the lack of guidance they typically receive. The extreme ambient temperatures and high relative humidity present in the Southeastern United States, but also in the more Southwestern region, mid-Atlantic region, and some parts of Europe, potentially pose a significant health risk to recreational athletes performing under these conditions. It is recommended that more educational initiatives be created by governing bodies in exercise and sports to inform the recreational running population on the benefits and proper implementation of an HA program.

## Figures and Tables

**Table 1:** Geographic distribution of participants (*N* = 125).

State	*n*	% of Sample
Alabama	52	41.6
Tennessee	35	28.0
Louisiana	19	15.2
Arkansas	12	9.6
Florida	3	2.4
Georgia	2	1.6
Mississippi	1	0.8
South Carolina	1	0.8

**Table 2:** Professional training guidance and HA information sources (*N* = 125).

* Professional	*n*	% of Sample
Running coach	18	14.4
Athletic trainer	4	3.2
Strength and conditioning specialist	4	3.2
Medical doctor	1	0.8
Other professional	7	5.6
No professional supervision	99	79.2

Note. * = Some participants reported receiving guidance from more than 1 type of professional/information source.

**Table 3:** M ± SD in Seasonal Training Practices for Runners of Low Training Status (*n* = 64) and High Training Status (*n* = 61).

	Summer			Fall		
	**Low**	**High**	***M*_Δ_**	**Low**	**High**	***M*_Δ_**
Duration (min.)	54.8 ± 18.4	68.8 ± 19.7	−14.0 *	61.0 ± 19.6	71.8 ± 18.8	−10.8 *
Frequency (days/wk)	4.0 ± 0.7	5.7 ± 0.9	−1.7 *	4.0 ± 0.9	5.7 ± 0.9	−1.7 *
km/wk	31.1 ± 9.2	64.7 ± 20.3	−33.6 *	35.2 ± 12.4	66.8 ± 21.6	−31.5 *
Rate of Perceived Exertion	6.3 ± 1.6	5.8 ± 1.5	0.5	5.9 ± 1.5	5.9 ± 1.4	0.0
min/wk	197.7 ± 67.1	381.6 ± 139.4	−183.9 *	218.6 ± 76.1	392.6 ± 141.0	−174.0 *
	**Winter**			**Spring**		
Duration (min.)	56.8 ± 17.8	68.1 ± 22.0	−11.4 *	58.6 ± 18.7	69.7 ± 20.6	−11.1 *
Frequency (days/wk)	3.8 ± 1.0	5.4 ± 1.2	−1.6 *	4.1 ± 0.8	5.7 ± 0.9	−1.6 *
km/wk	33.8 ± 14.6	63.4 ± 24.1	−29.5 *	35.4 ± 13.2	65.3 ± 20.1	−30.1 *
Rate of Perceived Exertion	5.6 ± 1.7	5.8 ± 1.5	−0.2	6.1 ± 1.5	6.1 ± 1.4	0.0
min/Wk	208.2 ± 81.2	363.3 ± 149.0	−155.1 *	214.6 ± 70.5	376.6 ± 140.5	−161.9 *

Note. *M*_Δ_ = mean difference; * denotes significant mean difference between the low and high groups based on *p* ≤ 0.05.

## Data Availability

Data from this study can be accessed by contacting the lead author, A.J.H, by email at alexander.heatherly@avemaria.edu.
